# Marriage and Mortality: The Importance of Non-Spousal Support for Never-Married Older Adults

**DOI:** 10.1093/geroni/igaa057.1114

**Published:** 2020-12-16

**Authors:** Adam Roth, Siyun Peng

**Affiliations:** Indiana University, Bloomington, Indiana, United States

## Abstract

Married individuals have been shown to consistently outlive their unmarried peers. Although numerous factors contribute to such mortality disparities, spousal support serves as one of the most central marital resources that reduces mortality risk for married older adults. Unmarried older adults, who lack access to such support, tend to rely more heavily on extended family and friends for their social needs. Yet it is unclear whether support from these non-spousal sources can be successfully substituted for spousal support to provide unmarried older adults with similar protection against mortality. In the present study, we use nationally representative data from the National Social Life, Health, and Aging Project to assess whether support from family and friends reduces the mortality differences between married and unmarried older adults. Although we examine all forms of singlehood (i.e., divorce, widowhood, and never married), we pay primary attention to never married older adults in relatively to their married peers as they have been exposed to a lifetime without a spouse. We find that never married older adults are especially sensitive to non-spousal support. More specifically, never married respondents who reported low levels of support were far more likely to die than married respondents who had similar levels of non-spousal support. Yet when support was high, never married respondents were neither more nor less likely to die than their married peers.

